# A Study in Red: The Overlooked Role of Azo‐Moieties in Polymeric Carbon Nitride Photocatalysts with Strongly Extended Optical Absorption

**DOI:** 10.1002/chem.202102945

**Published:** 2021-10-21

**Authors:** Dariusz Mitoraj, Igor Krivtsov, Chunyu Li, Ashwene Rajagopal, Changbin Im, Christiane Adler, Kerstin Köble, Olena Khainakova, Julian Hniopek, Christof Neumann, Andrey Turchanin, Ivan da Silva, Michael Schmitt, Robert Leiter, Tibor Lehnert, Jürgen Popp, Ute Kaiser, Timo Jacob, Carsten Streb, Benjamin Dietzek, Radim Beranek

**Affiliations:** ^1^ Institute of Electrochemistry Chemistry Ulm University Albert-Einstein-Allee 47 89081 Ulm Germany; ^2^ Department of Organic and Inorganic Chemistry University of Oviedo 33006 Oviedo Spain; ^3^ Institute of Physical Chemistry and Abbe Center of Photonics Friedrich Schiller University Jena Lessingstr. 10 07743 Jena Germany; ^4^ Department Functional Interfaces Leibniz Institute of Photonic Technology (IPHT) Albert-Einstein-Str. 9 07745 Jena Germany; ^5^ Institute of Inorganic Chemistry I Ulm University Albert-Einstein-Allee 11 89081 Ulm Germany; ^6^ Department Spectroscopy/Imaging Leibniz Institute of Photonic Technology (IPHT) Albert-Einstein-Str. 9 07745 Jena Germany; ^7^ Center for Energy and Environmental Chemistry Jena (CEEC Jena) Philosophenweg 7a 07743 Jena Germany; ^8^ ISIS Neutron and Muon Source Rutherford Appleton Laboratory Harwell Oxford Didcot OX11 0QX UK; ^9^ Electron Microscopy of Materials Science Central Facility for Electron Microscopy Ulm University Albert-Einstein-Allee 11 89081 Ulm Germany; ^10^ Helmholtz-Institute-Ulm (HIU) Helmholtzstr. 11 89081 Ulm Germany; ^11^ Karlsruhe Institute of Technology (KIT) P.O. Box 3640 76021 Karlsruhe Germany

**Keywords:** carbon nitrides, hydrogen, photocatalysis, solar energy conversion, visible light

## Abstract

The unique optical and photoredox properties of heptazine‐based polymeric carbon nitride (PCN) materials make them promising semiconductors for driving various productive photocatalytic conversions. However, their typical absorption onset at ca. 430–450 nm is still far from optimum for efficient sunlight harvesting. Despite many reports of successful attempts to extend the light absorption range of PCNs, the determination of the structural features responsible for the red shift of the light absorption edge beyond 450 nm has often been obstructed by the highly disordered structure of PCNs and/or low content of the moieties responsible for changes in optical and electronic properties. In this work, we implement a high‐temperature (900 °C) treatment procedure for turning the conventional melamine‐derived yellow PCN into a red carbon nitride. This approach preserves the typical PCN structure but incorporates a new functionality that promotes visible light absorption. A detailed characterization of the prepared material reveals that partial heptazine fragmentation accompanied by de‐ammonification leads to the formation of azo‐groups in the red PCN, a chromophore moiety whose role in shifting the optical absorption edge of PCNs has been overlooked so far. These azo moieties can be activated under visible‐light (470 nm) for H_2_ evolution even without any additional co‐catalyst, but are also responsible for enhanced charge‐trapping and radiative recombination, as shown by spectroscopic studies.

## Graphical abstract

**What makes it red**? A novel high‐temperature treatment procedure for turning the conventional melamine‐derived yellow polymeric carbon nitride into a red carbon nitride is reported. Surprisingly, a detailed characterization reveals that the red colour comes from the azo‐groups formed during the synthesis, a moiety whose role in shifting the optical absorption edge and photocatalytic activity of carbon nitrides more into the visible range has been overlooked so far.

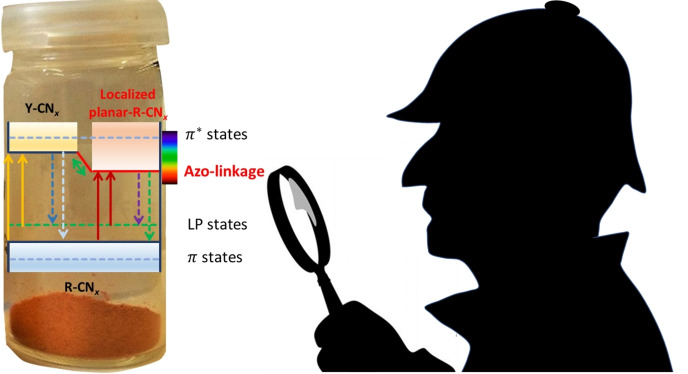

## Introduction

Heptazine‐based polymeric carbon nitride (PCN) materials^[1]^ stand out from a range of other typical, mostly metal oxide‐based photocatalysts mainly by their unique optical (i. e., bandgap and intra‐gap states)^[2]^ and photoredox (i. e., *quasi*‐Fermi levels of electrons and holes) properties.^[3]^ Nevertheless, the typical absorption onset of PCN is around 430–450 nm, which is far from making it an ideal absorber of solar radiation. This motivated significant research efforts directed towards shifting the optical absorption and the corresponding photocatalytic activity to the red. In general, one can distinguish two basic strategies for achieving this goal. Firstly, modified synthetic protocols aim at incorporating additional light absorbing moieties already during the synthesis of PCN, for example, by introducing sulfur species,^[4]^ aromatic carbon moieties,^[5]^ creating co‐polymeric precursors with other N‐containing molecules,^[6]^ or by varying the condensation conditions, especially the temperature^[7]^ or reaction medium composition.^[8]^ Secondly, post‐synthetic approaches comprise chemical surface modification of conventional PCN, for instance, by hydrogenation or oxidation,^[9]^ sensitization with light harvesting compounds such as organic dyes,^[10]^ carbon dots or fullerenes,^[11]^ or various forms of post‐synthetic thermal treatment.^[12]^

Although the condensation of PCN precursors at high temperatures (above 600 °C)^[7]^ and the post‐synthetic annealing of PCNs under similar conditions^[12]^ might seem alike, the essential difference lies in the lower ammonia gas evolution from the material in the latter case. While the two approaches have somewhat similar effects on the material's optical properties if carried out under inert or reductive (NH_3_, H_2_) conditions[^7^, ^13^] or under vacuum,^[14]^ the reports on the performance of the resulting photocatalysts in terms of activity or visible‐light response are contradictory and show little consensus regarding the optimal thermal treatment conditions. For example, Wang et al. reported a continuous increase of the photocatalytic H_2_ evolution rate with increasing temperature of PCN condensation up to 700 °C.^[12]^ In accord with this result, P. Niu et al. demonstrated an activity enhancement of PCN post‐treated at 750 °C.^[15]^ Nevertheless, other authors observed a maximum activity at intermediate heat‐treatment (condensation or post‐treatment) temperatures of 500 °C,^[14]^ 550 °C,^[7]^ and 580 °C,^[16]^ 600 °C^[10]^ or 680 °C,^[17]^ followed by a significant drop in photocatalytic activity. These discrepancies in the reported photocatalytic performance of PCNs with extended visible‐light absorption are clearly due to the lack of our knowledge on the nature of the colour centres that are responsible for the activation beyond wavelengths of ∼450 nm in various PCNs. A number of plausible suggestions have been made to explain the red shift of the optical absorption edge, including increased condensation degree due to a partial residual NH_2_‐groups removal,^[7]^ improved stacking allowing normally forbidden n‐π* transitions,^[12]^ and creation of N‐[^17^, ^18^] or C‐vacancies.[^13a^, ^19^] Nonetheless, given the disordered nature of PCN polymers, it is difficult to unambiguously prove the distinct type of colour centres in the material, so that they often remain rather hypothesised than proven. However, the determination of the moieties or structural features responsible for the red shift is crucial for developing an appropriate synthetic or post‐synthetic technique for the preparation of efficient visible‐light active PCNs.

Herein, we report a post‐synthetic approach involving a brief (3 min) high‐temperature (above 900 °C) treatment under vacuum that effectively turns the typically yellow PCN into a red PCN by significantly extending its visible light absorption down to >600 nm. Interestingly, we demonstrate that the dramatic shift of the absorption edge is due to the formation of azo‐linkages in the PCN structure, an aspect that has been largely overlooked so far. Finally, we elucidate and discuss the effects of the azo moieties on the charge transfer dynamics and photocatalytic activity of the obtained photocatalysts. This sheds some critical light on the general applicability of PCN photocatalysts with drastically extended visible‐light absorption prepared by methods comprising a high‐temperature treatment.

## Materials and Methods

**Synthesis of polymeric carbon nitride**: The sample of conventional yellow PCN (Y‐CN_x_) was prepared by thermal polycondensation of 30 g melamine at 530 °C for 4 h in a lid‐covered crucible. The red PCN materials (R‐CN_x_) were prepared by a heat treatment of Y‐CN_x_ in a quartz tube initially having a reduced pressure of 1.8×10^−2^ mbar using a tube furnace operating at 900 °C for 3 min. After the treatment the samples were cooled down, brought to ambient pressure and powdered.

The dissolution of the synthesized samples was carried out by a modified method proposed by Horvarth‐Bordon et al.^[20]^ 1.0 g of the Y‐CN_x_ or R‐CN_x_ samples was suspended in 50 mL of 1 M aqueous KOH solution and transferred into a Teflon‐lined stainless steel autoclave with a total volume of 100 mL. The autoclaves were heated at 100 °C for 16 h, then the obtained solution was filtered first through a paper filter and then through a 0.25 μm PES syringe filter. Tri‐potassium cyamelurate crystals were produced from the above‐mentioned solutions by evaporation and precipitation. The needle shaped tri‐potassium cyamelurate crystals formed in both cases were filtered, washed with ethanol and dried. In order to analyse the presence of minor products of dissolution in KOH of Y‐CN_x_ (Y‐CN_x_‐K) and R‐CN_x_ (R‐CN_x_‐K), the solutions were subjected to several cycles of evaporation, tri‐potassium cyamelurate crystals separation and subsequent concentration of the supernatant. The supernatant was subsequently analysed. The solid crystalline powder was obtained from the R‐CN_x_‐K solution (R‐CN_x_‐KS) by slow evaporation of water from the R‐CN_x_‐K solution, then it was washed with deionized water and dried overnight at 70 °C.

**Deposition of co‐catalysts for photocatalytic H_2_ reactions**: The preparation of photocatalysts modified with Pt nanoparticles or [Mo_3_S_13_]^2−^ (={Mo_3_}) clusters was described in detail in our previous work.^[21]^ Briefly, the platinized Y‐CN_x_ and R‐CN_x_ samples were prepared using photodeposition method. For this, the Y/R‐CN_x_ powder (0.2 g) was suspended in 20 mL H_2_O:MeOH (9 : 1, v : v) containing hexachloroplatinic acid (4 mg), ultrasonicated for 1 h, bubbled with argon gas and irradiated for 30 min with a 150 W Xe‐lamp equipped with a KG3 (Schott) heat filter (intensity ca. 1 sun). Subsequently, the powder was washed by centrifugation and dried overnight at 60 °C.

The {Mo_3_} clusters were synthesized using (NH_4_)_2_[Mo_3_S_13_]×2H_2_O produced by the method described by Müller et al.[^21^, ^22^] Then Na_2_[Mo_3_S_13_]×5H_2_O (={Mo_3_}) was obtained from (NH_4_)_2_[Mo_3_S_13_]×2H_2_O by its reaction in aqueous NaOH solution under reduced pressure (20 mbar) for 2 h. The resulting dark red solution was filtered into an aqueous NaCl solution, the precipitated product was isolated by filtration, washed with 2‐propanol and diethyl ether, and dried *in vacuo* at room temperature to give red‐brown {Mo_3_}. The Y‐CN_x_‐{Mo_3_} and R‐CN_x_‐{Mo_3_} hybrids were synthesized through a two‐step deposition process at room temperature. In a typical procedure, 20 mg of as‐prepared Y/R‐CN_x_ was dispersed in 20 mL of methanol by ultrasonication for 3 h to prepare a homogenous suspension. Then, a measured amount of {Mo_3_} (soluble in methanol) was added and stirred for 24 h. The solid was collected by centrifugation and three times washed with methanol to remove the excess of {Mo_3_}. Finally, the obtained precipitates were dried at room temperature under ambient conditions yielding Y‐CN_x_‐{Mo_3_} and R‐CN_x_‐{Mo_3_}.

## Characterization

**FTIR spectroscopy** was performed on a Shimadzu FTIR‐8400S spectrometer. Samples were prepared as KBr pellets. X‐ray powder diffraction (XRD) data were collected on a Rigaku XRD‐6000 and a Pananalytical X'pert PRO diffractometers using Cu Kα radiation (λ=0.154 nm). **X‐ray photoelectron spectroscopy (XPS)** measurements were performed from the samples deposited on a gold support using a UHV Multiprobe system (Scienta Omicron, Germany) with a monochromatic X‐ray source (Al K_α_) and an electron analyzer (Argus CU) with 0.6 eV energy resolution. Charge compensation during data acquisition was realized by an electron flood gun (NEK 150SC, Staib, Germany) at 6 eV and 50 μA. The background was subtracted and spectra were calibrated using the Au 4f_7/2_ peak (84.0 eV) before undergoing fitting using Voigt functions (30 : 70).

**UV Raman spectroscopy** was performed on a custom modified (to allow UV excitation) LabRAM HR 800 Raman System (Horiba Jobin Yvon, Bensheim, Germany) with an excitation wavelength of 244 nm provided by frequency doubling the 488 nm line of an Ar‐Ion‐Laser (Innova 300 C Moto‐FRED, Coherent Inc., Santa Clara, CA, United States of America). The laser power was attenuated from 13 mW to 5 mW or 10 mW via a neutral density filter depending on the photothermal stability of the samples. The samples were illuminated through a microscope (BX 41, Olympus Corporation, Tokyo, Japan) through a 15×/0.2NA UV objective (LMU UVB, Thorlabs Inc., Newton, NJ, United States of America) with UV optimized antireflection coating. The scattered light is collected in 180° geometry via the same objective, filtered through two long‐pass filters to remove the Rayleigh scattered light, passed through an entrance slit (width=300 μm) and diffracted by a 2400 lines/mm blazed grating to a liquid N_2_‐cooled CCD (T_op_=160 K). All spectra were recorded with an integration time of 300 s and 12 averaged accumulations (i. e., 1 h of summed integration time). All samples were measured as manually compacted powders in a small stainless‐steel mold. The Raman spectra were processed using R 3.5.1 by applying SNIP based baseline correction (100 iterations) and normalizing the spectra using Euclidean vector norm.^[23]^

**FT Raman spectroscopy** was performed on a Multispec FT‐Raman spectrometer (Bruker Inc., Billerica, Massachusetts, United States of America). The excitation light at 1064 nm was provided by a Nd:YAG laser (Klastech DeniCAFC‐LC‐3/40, Dortmund, Germany). The spectra were recorded up to 4000 cm^−1^ with a spectral resolution of 4 cm^−1^. The laser power at the samples was set to 200 mW for R‐CN_x_ and 500 mW for Y‐CN_x._ R‐CN_x_‐KS could not be measured using FT‐Raman spectroscopy due to excessive photoluminescence.

The Raman spectra were processed using R 3.5.1 by applying SNIP based baseline correction (60 iterations) and normalizing the spectra to the ring‐breathing mode at 980 cm^−1^ for visualization.

**Neutron total scattering measurements**: Time‐of‐flight powder neutron diffraction experiments were conducted using the GEM diffractometer at the ISIS pulsed neutron and muon source, Rutherford Appleton Laboratory, UK.^[24]^ Powder samples of Y‐CN_x_ and R‐CN_x_ were loaded into 6 mm diameter cylindrical vanadium sample holders. Data collection consisted of ∼ 6 h duration (1000 μA h) data sets, acquired at 300 K for total scattering analysis. MantidPlot software^[25]^ was used for data reduction and normalisation. Total scattering data (covering a scattering range 0.5<*Q*<50 Å^−1^ when using the final processed files from the six GEM detector banks) were corrected and the pair distribution function was generated using the GUDRUN software.^[26]^

**Transmission electron microscopy (TEM)**: The samples were dispersed in ethanol (supersonic bath) and drop‐cast on holey carbon TEM grids. The TEM investigations were carried out using the chromatic (Cc) and spherical (Cs) aberration ‐ corrected Sub‐Ångström Low‐Voltage Electron (SALVE) microscope operating at 80 kV. Values for Cc and Cs were in the range of −10 μm to −20 μm. Imaging included diffraction pattern from selected areas and high resolution (HR) TEM, recorded on a Ceta 16 M camera. Energy‐filtered imaging exploiting absorption edges for elemental mapping was acquired on an Ultrascan 1000 XP camera using a Quantum ERS low‐voltage Gatan imaging filter (GIF), attached to the SALVE microscope.

**Diffuse reflectance spectroscopy (DRS) UV‐vis spectra** of solids were taken by a Shimadzu UV2600 UV‐vis spectrophotometer. **UV‐vis spectra** of the dissolved in KOH carbon nitride samples were recorded using a Cary 60 (Agilent Technologies) spectrophotometer.

**Thermal decomposition** of the carbon nitride samples was studied using a Mettler Toledo TGA/SDTA 851e at a heating rate of 10 °C min^−1^ in O_2_ flow (50 mL min^−1^) from 30 °C to 850 °C.

**CHN analysis** was performed using an Elementar Vario MICRO cube.

The EDX analysis was carried out using a Scanning electron microscope (Supra 55VP, SmartSEMTM, Zeiss) equipped with an Oxford Instruments EDX detector.

The solid‐state ^1^H, ^13^C, MAS NMR, ^1^H‐^13^C and ^1^H‐^15^N CPMAS NMR spectra were registered at the spinning rates of 12 kHz for ^1^H and 5 kHz for all other measurements using a Bruker Avance III 400WB spectrometer. ^1^H, ^13^C and ^15^N chemical shifts are quoted in ppm from: TMS (0 ppm) and α‐glycine (secondary reference, C=O at 176.0 ppm and NH_3_^+^ at 347.6 ppm on the nitromethane scale, respectively).

**Liquid state** ^**13**^ **C NMR** characterization of the products of the dissolution of carbon nitride samples in aqueous KOH (1.0 M) was carried out in a Bruker Avance II 400 MHz spectrometer at a 100 MHz frequency using TMS (0 ppm) standard. The samples were diluted in D_2_O prior to analysis.

**Gas chromatography**: Gas‐chromatography was performed on a Bruker Scion GC/MS, with a thermal conductivity detector 15 (column: molecular sieve 5 A 75 m×0.53 mm, oven temperature 70 °C, flow rate 25 ml min ^−1^, detector temperature 200 °C) with Argon as carrier gas. The GC was calibrated by direct injection of known amounts of H_2_ gas.

**Emission spectroscopy**: Polymeric carbon nitride suspensions for spectroscopic study were prepared by sonicating the carbon nitrides in deionized water (2 mg/mL) for 15 min. The supernatant liquid was collected after centrifugation (10 min, 7500 rpm) and used for steady‐state emission and time‐ressolved emission spectroscopy. Steady‐state emission spectra (λ_ex_=380 nm) were recorded with a FLS980 spectrometer (Edinburgh Instruments) in a 1 cm quartz cell. Time resolved emission data were recorded using a Hamamatsu HPDTA streak camera. A Ti:sapphire laser (Tsunami, Newport Spectra‐Physics GmbH) was used as the light source and a pulse selector (model 3980, Newport Spectra‐Physics GmbH) was used to reduce the repetition rate of the fundamental to 400 kHz. The emission was collected from a 1 cm cuvette in a 90° angle between the pump beam and a CHROMEX spectrograph detector. Analysis of the time‐resolved emission data was performed using DecayFit software.

### Photocatalytic studies

The photocatalytic hydrogen production experiments were carried out using a custom‐built air‐cooling apparatus for maintaining room temperature (22 °C) and constant irradiation of the sample. Experiments were carried out in 21 mL Schlenk tubes capped with rubber septa. 10 mg of the CN_x_‐based catalysts, which had been found to be the optimal loading determined in our previous study,^[21]^ was added to the Schlenk tube equipped with magnetic stirrer. The tube was evacuated and filled with argon. An H_2_O : MeOH (9 : 1, v/v) solution, with or without addition of additional sacrificial electron donor, was purged with argon. 10 mL of the degassed solution was added to the photocatalyst in the Schlenk tube under inert conditions and kept under constant irradiation from an LED source (λ=420 nm; 51.3 mW cm^−2^ or λ=470 nm; ca. 40 mW cm^−2^) in air‐cooled photoreactors. The gas phase above the solution was probed by inserting a gas‐tight GC syringe through the septum and the amount of hydrogen in the gas phase was quantified using headspace gas chromatography.

### Computational studies

All periodic density functional theory (DFT) calculations were performed with the Vienna Ab initio Simulation Package (VASP 5.4.4),^[27]^ utilizing the projector‐augmented wave (PAW) method to describe the core electrons, while valence states were expanded by plane waves up to an energy cut‐off of 400 eV. Geometries were pre‐optimized (up to a force criterion of 5 ⋅ 10 ^−2^ eV ⋅ Å ^−1^) with the Perdew ‐Burke ‐Ernzerhof (PBE)^[28]^ generalized‐gradient‐approximation (GGA) exchange‐correlation functional, followed by a fine‐optimization using the Heyd ‐Scuseria ‐Ernzerhof (HSE06)^[29]^ hybrid functional (with the range separation parameter of 0.2 Å^−1^) and 25 % exact exchange. Van der Waals interactions were treated with Grimme's D3 dispersion correction scheme.^[30]^ Self‐consistency in the electronic structures were set to a limit of 10^−6^ eV, utilizing a Gaussian smearing of 0.01 eV. Periodic images of the two‐dimensional systems were separated by a vacuum layer of at least 20 Å, together with dipole corrections. Integrations in the reciprocal space were evaluated on a Monkhorst‐Pack^[31]^ grid of 5×5×1 *k*‐points.

## Results and Discussion

### Materials characterization

**Figure 1:**
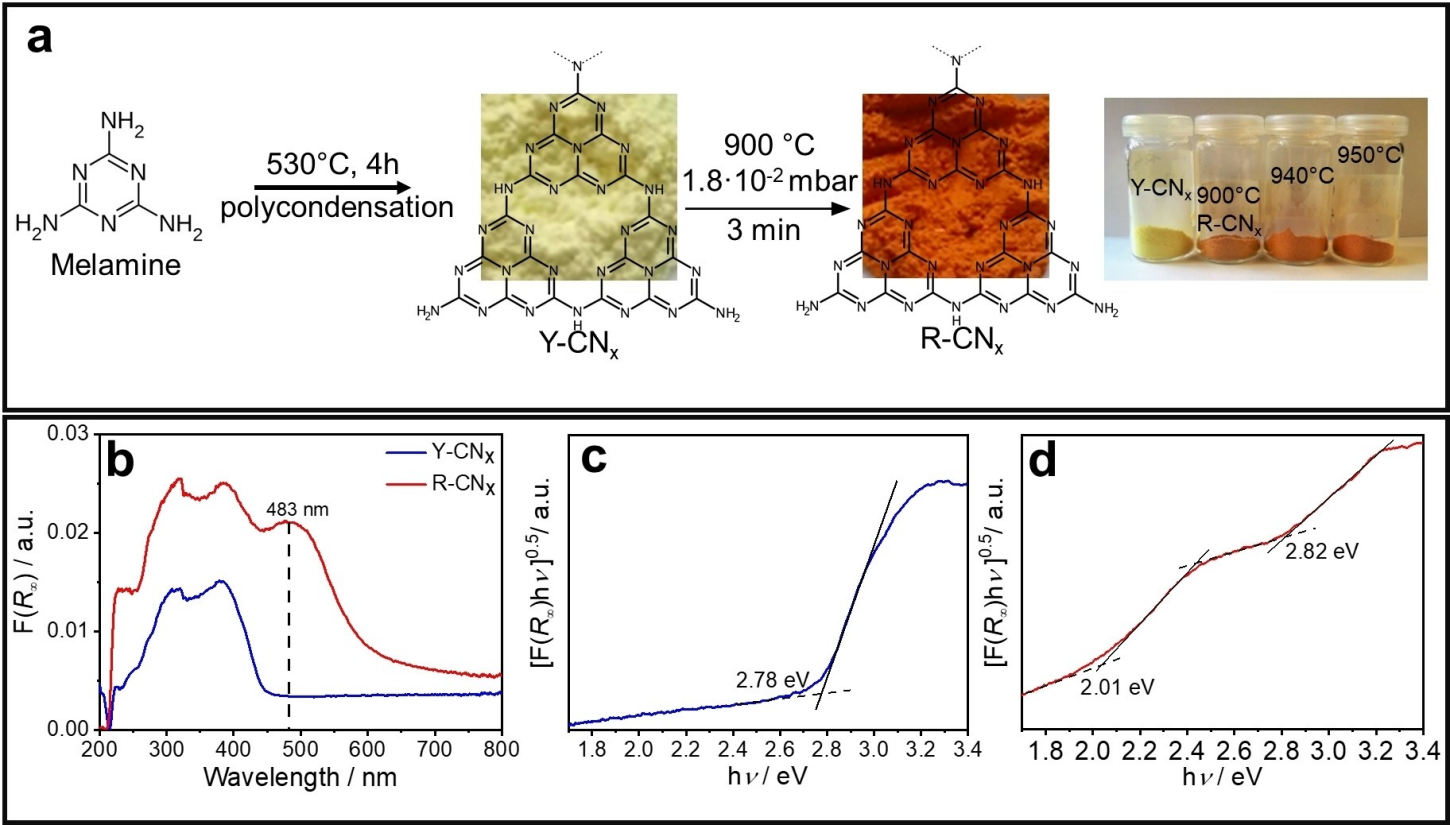
(a) Schematic representation of the synthetic procedures for conventional yellow Y‐CN_x_ and low‐bandgap reddish R‐CN_x_; diffuse reflectance spectra (b) and corresponding Tauc plots (assuming direct nature of the optical transition) for Y‐CN_x_ (c) and R‐CN_x_ (d).

A schematic representation of the synthetic procedures implemented in this work is depicted in Figure 1a. The change of colour from yellow to nearly red is observed if Y‐CN_x_ is heated at temperatures above 900 °C for a brief time interval (3 min) and under reduced pressure. The increase of the thermal treatment temperature or prolongation of this procedure do not lead to any further observable changes of the material's appearance and only result in lower yields (Figure 1a). The Y‐CN_x_ sample has a typical UV‐vis diffuse reflectance spectrum (Figure 1b) with an absorption edge of around 2.9 eV (Figure 1c), while R‐CN_x_ shows another visible‐light absorption band centred at ca. 483 nm (Figure 1b). Apparently, the new absorption peak in the visible region observed for the R‐CN_x_ sample (Figure 1b) is an additional feature and can hardly be considered as shifted Y‐CN_x_ absorption edge, which remains visible and manifests itself at an electronic transition energy of 2.9 eV, while the new contribution has an onset at ca. 2.2 eV (Figure 1d).

Notably, R‐CN_x_ and Y‐CN_x_ possess very similar elemental composition of C_2.88_N_4.19_H_1.69_ and C_2.89_N_4.28_H_1.84_, respectively, with only a small reduction of N and H content in the red materials as compared to the yellow one. The thermal behaviour of the both samples is also alike, the R‐CN_x_ sample, though showing slightly lower degradation onset, reaches the maximum decomposition rate ca. 30 °C higher than Y‐CN_x_ (see Supporting Information Figure S1). As evidenced by TEM, the microstructure and morphology of the Y‐CN_x_ and R‐CN_x_ samples are also practically identical, thus not allowing to draw any definite conclusions regarding the high‐temperature induced modifications (see Supporting Information Figure S7).

**Figure 2:**
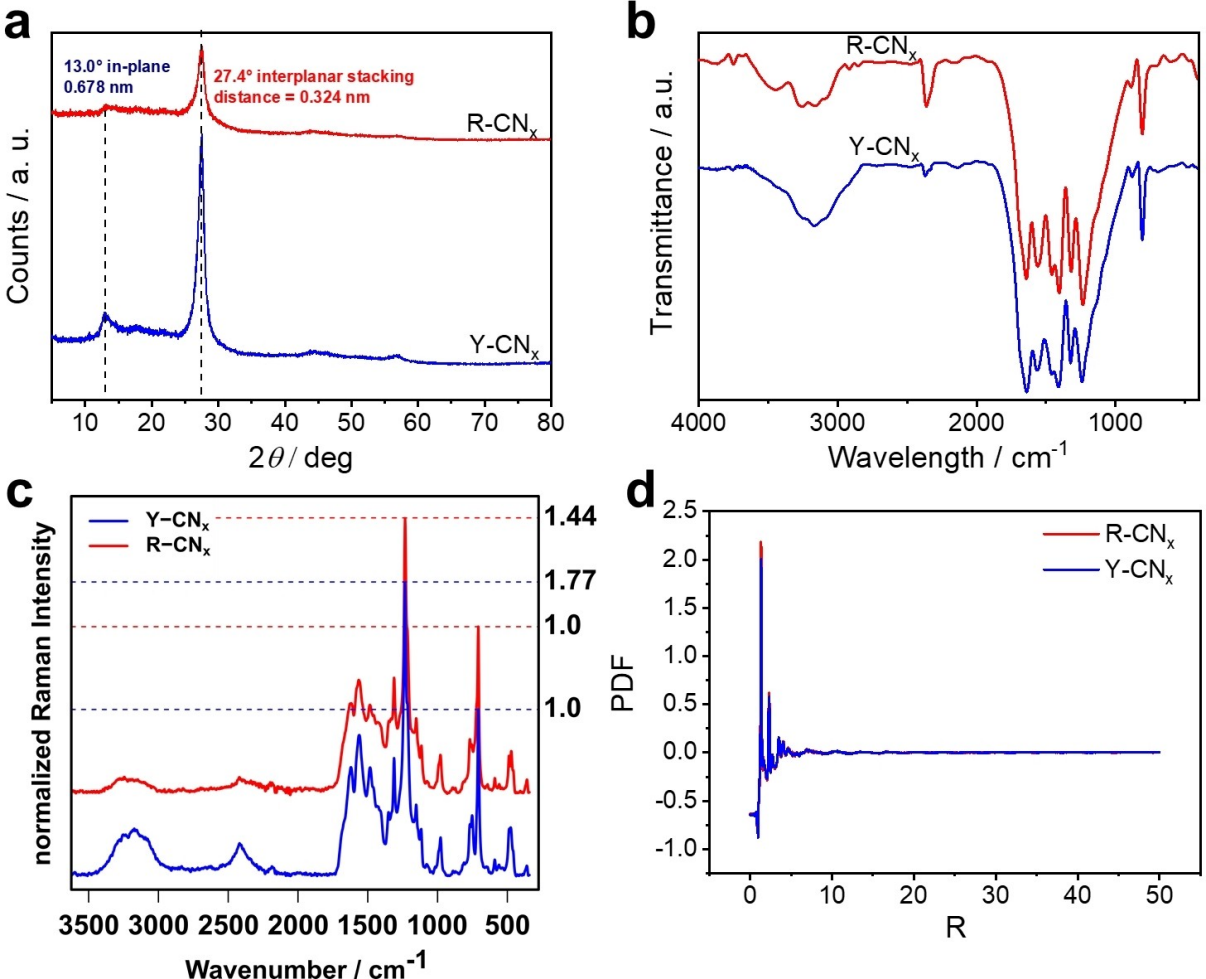
(a) powder XRD, (b) FTIR, (c) Raman and (d) neutron diffraction PDF calculated data obtained for Y‐CN_x_ and R‐CN_x_.

XRD data manifest that the typical PCN structure persists in the R‐CN_x_ material also after the high temperature treatment (Figure 2a). The reflections corresponding to the typical for PCN (100), (002) and even the less intense (300) and (004) planes^[32]^ are clearly distinguishable in the R‐CN_x_ diffractogram, although having somewhat lower intensity owing to partial amorphization of PCN under thermal treatment at 900 °C (Figure 2a). FTIR data also point to similar structure of Y‐CN_x_ and R‐CN_x_ as all the typical bands characteristic of PCN are also found in the spectrum of R‐CN_x_ (Figure 2b), which is not the case, for instance, when PCN is treated in NaCl melt at high temperatures.^[8]^ Although no definite conclusion can be made from it, the appearance of another band at around 3400 cm^−1^ in the spectrum of R‐CN_x_ suggests the modification of H‐bonding in the treated material (Figure 2b). The FT‐Raman spectra of these two materials confirm the observations obtained by the IR‐ and XRD‐measurements. According to the Raman spectra, the structure of Y‐CN_x_ and R‐CN_x_ are practically the same with only minor changes in intensity ratios in the fingerprint region (Figure 2c). The most noticeable difference between the two spectra is the stark decrease of intensity of the broad band between 2300 and 2500 cm^−1^ in R‐CN_x_. This band can be assigned to a second‐order vibration of graphite‐like carbon nitride and can only be observed in ordered structures, which supports the XRD‐observed amorphization of the PCN.^[33]^ The band at 806 cm^−1^ can be assigned to the ring‐breathing mode of the triazines present in the CN_x_ structure while the band at 1235 cm^−1^ is specific to the ν(C−N) stretching vibration of the bridging amine moieties in the heptazine structure.^[34]^ FT‐Raman spectroscopy shows that the ratio of bridging amine to the sym‐triazine breathing mode decreases in R‐CN_x_ when comparing to Y‐CN_x_. This could indicate a replacement of the bridging amine moieties by some other bridging functionality, which is further supported by the intensity increase of the shoulder located at 1662 cm^−1^ relative to the main band at 1618 cm^−1^ (aromatic ring stretching). This increase in intensity could indicate an increase in *exo* unsaturated functionalities. Due to the complicated spectra and the possible loss of unpolymerized triazine impurities during the heat‐treatment, the Raman spectra do not allow for definitive conclusions regarding the precise chemical structure, but can at least confirm changes to the binding conditions and offer a hint that amine bridges are replaced by unsaturated functionalities. Neutron diffraction, possibly the most adequate tool for structural characterization of carbon nitrides composed of low electron density atoms,^[32]^ was further employed as a sensitive technique to detect the structural changes in PCN caused by the thermal treatment. The structure function F(Q) (Figure S3) as well as the calculated pair distribution function (PDF) data (Figure 2d) obtained from the neutron diffraction, however, show no significant differences in the analysed samples. This suggests that the PCN structure is mostly preserved in the R‐CN_x_ material and no significant changes of the interlayer distances corresponding to the corrugation or planarization can be evidenced, which were previously suggested to be responsible for the colour change.[^7^, ^12^]

The C 1s XP spectra for both materials are dominated by the peak at 283.8 eV attributed to adventitious carbon, while the maxima at 285.7, 287.3 and 290.1 eV are assigned to C‐NH_x_, N=C−N, and COOH species respectively (Figure S4). The N 1s spectrum of Y‐CN_x_ can be fitted with four peaks having their maxima at binding energy values typical of PCN 397.8, 399.7, 401.5 and 404.1 eV, the latter one being ascribed to π‐π excitation in the aromatic polyheptazine system (Figure S5a). The peak at 401.5 eV corresponds to the nitrogen in C‐NH_x_ groups^[35]^ and the maximum 397.8 eV is attributed to the C−N=C aromatic species. The contribution centred at 399.7 eV, usually assigned to N−C_3_ groups,^[36]^ should be rather assigned to the sum of these species and the triazine impurities, since the ratio between the maxima at 397.8 and 399.7 eV is far below 6, the value one might expect to obtain for the heptazine structure. We assume that the Y‐CN_x_ sample contains certain amount of non‐condensed impurities, which is in agreement with the XPS data obtained for melamine, which shows equal contributions at 398.4 and 399.7 eV in its XP N 1s spectrum.^[37]^ The respective spectrum of the R‐CN_x_ sample has, on the other hand, a more conventional profile, where the peaks corresponding to C−N=C, N−C_3_ and C−NH_x_ are clearly distinguished at 398.1, 399.5 and 400.5 eV respectively (Figure S5b).

**Figure 3:**
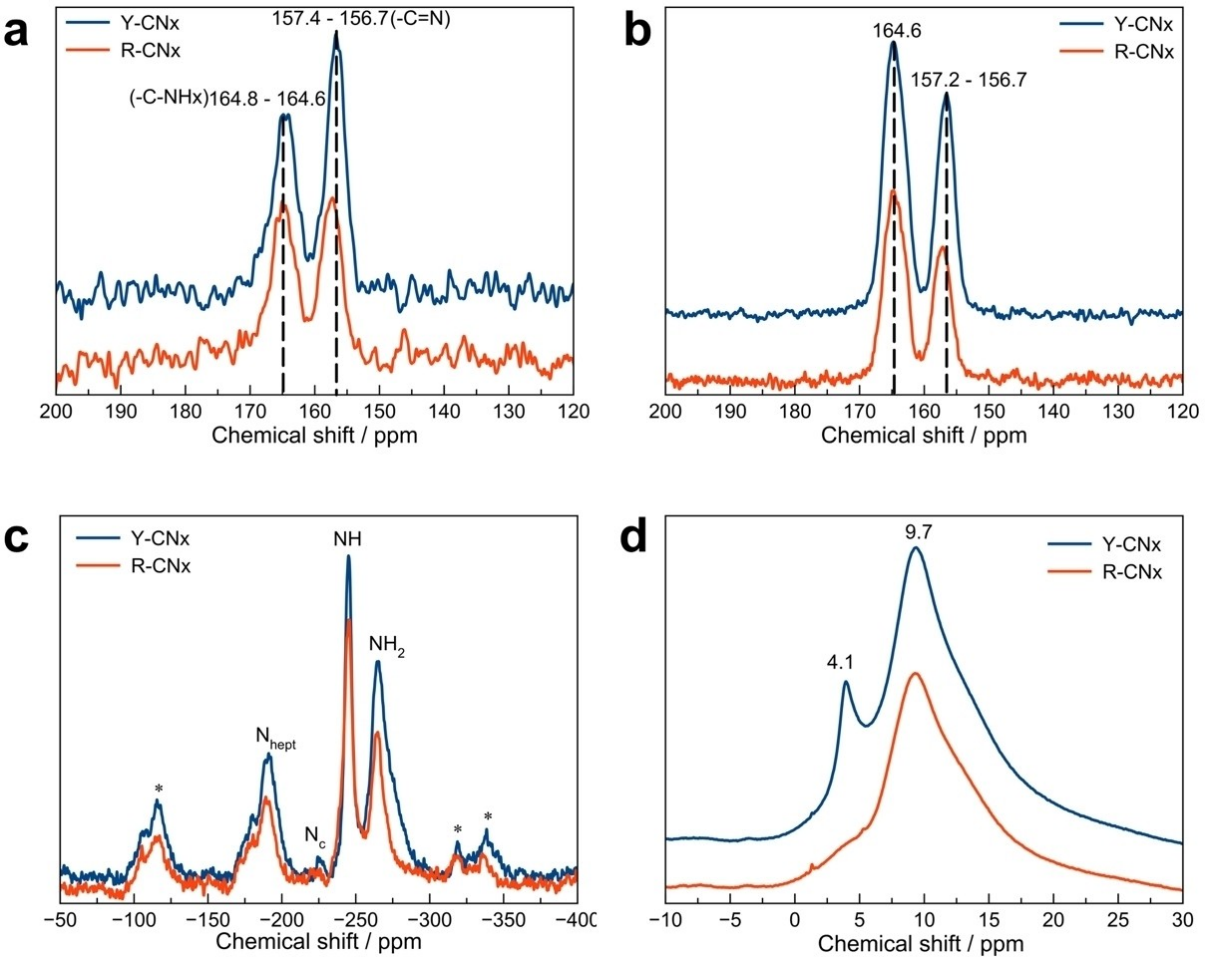
SS NMR (a) ^13^C MAS, (b) ^1^H‐^13^C CPMAS, (c) ^1^H‐^15^N CPMAS and (d) ^1^H MAS spectra of Y‐CN_x_ and R‐CN_x_.

The direct ^13^C NMR excitation spectra show a typical profile for heptazine based PCN materials with maxima in the range of 157–156 ppm and 165–164 ppm corresponding to the carbon atoms in N−C=N and −C−NH_x_ groups, respectively (Figure 3a). The reduction of the peak intensity and its slight shift from 156.7 to 157.4 ppm with respect to that of Y‐CN_x_ is evident in the spectrum of R‐CN_x_ (Figure 3a). This shift might be attributed either to a higher degree of disorder due to heptazine fragmentation in R‐CN_x_ caused by the thermal treatment or to the formation of some new species in the material. According to the tabulated data of ^13^C NMR chemical shifts,^[38]^ the appearance of imine‐like or azomethine groups (>C=N−) formed as the result of heptazine fragmentation is one of the conceivable possibilities. Another possibility is the formation of −C−N=N−C− azo functions that are known to promote visible‐light absorption of tuned carbon nitrides.^[39]^ The ratio between the peaks assigned to N−C=N to that of C−NH_x_ in the ^13^C spectra is around 1.1 for Y‐CN_x_ and 0.8 for R‐CN_x_. We assume that this might be attributed to a partial fragmentation of the heptazine structure in the thermally treated material, hence the loss of aromatic carbon‐species. The ^1^H‐^13^C CPMAS spectra show the enhancement of the peak intensity at 165–164 ppm confirming its assignment to the C‐NH_x_ moieties (Figure 3b). The intensity of the peak at around 157.4 ppm in the spectrum of R‐CN_x_ is not increased significantly, suggesting that the new species formed after the treatment is not in the immediate proximity to any H atoms. This leads us to the conclusion that the formation of −C−N=N−C− azo linkages is more likely (Figure 3b). The ^1^H‐^15^N CPMAS spectra show the same set of peaks at −191, −224, −245 and −265 ppm for both samples, which can be assigned to the peripheral heptazine N atoms, central heptazine nitrogen, nitrogen of NH− groups and of NH_2_ species, respectively (Figure 3c).^[35]^ A reduction of the FWHM and the intensity of the peak ascribed to the NH_2_ functions can be observed for R‐CN_x_ compared to Y‐CN_x_ (Figure 3c). The peak fitting shows that the contribution centred at about −274 ppm is significantly reduced in the case of the R‐CN_x_ sample (Figure S6). The amino‐groups can manifest themselves in a large range of chemical shifts from −267 to −291^[40]^ depending on their environment. Thus, we assume that the reduction of the NH_2_ peak contribution at −274 ppm might be due to the elimination of more volatile carbon nitride constituents or impurities during the thermal treatment. The ^1^H spectra are composed of two main contributions at 9.7 and 4.1 ppm which correspond to the hydrogens of hydrogen‐bonded amino‐groups and adsorbed water molecules, respectively (Figure 3d).^[41]^

**Figure 4:**
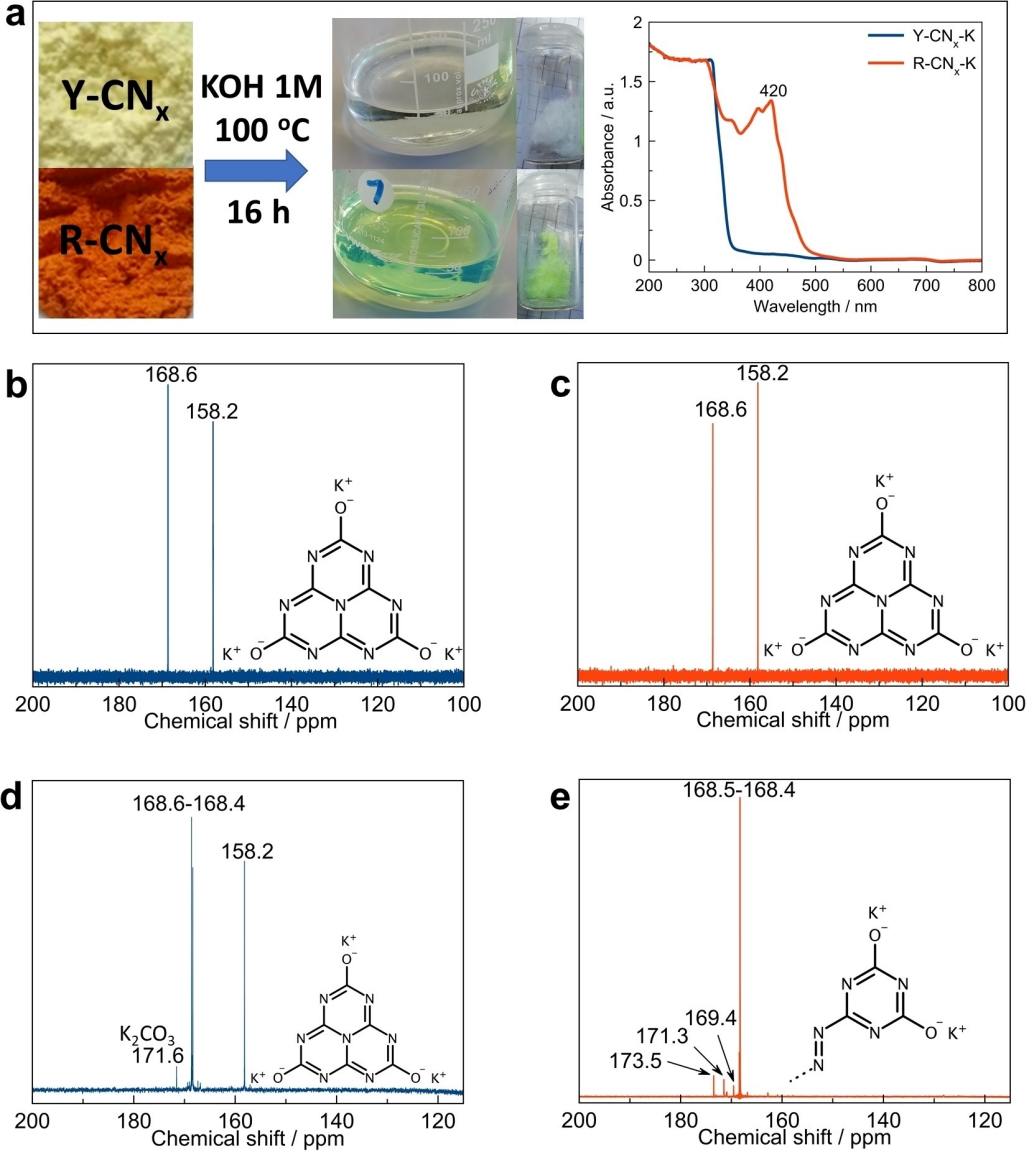
(a) The products of the Y‐CN_x_ and R‐CN_x_ dissolution in KOH and their UV‐vis spectra; ^13^C NMR spectra in D_2_O of the crystals obtained from the dissolved in KOH (b) Y‐CN_x_ and (c) R‐CN_x_ samples; ^13^C NMR spectra of the minor products of the (d) Y‐CN_x_ and (e) R‐CN_x_ samples dissolution in KOH with addition of D_2_O.

The comparative analysis of R‐CN_x_ and Y‐CN_x_ presented above shows that the structural modifications produced by the high‐temperature treatment are not easily detectable by conventional solid‐state characterization approaches, although they clearly have a great impact on the light absorption properties of the materials (Figure 1). Therefore, further attempts to characterize the R‐CN_x_ material were carried out starting by its dissolution in an appropriate solvent. The dissolution of PCN in KOH proceeds via hydroxyl attack on amino‐groups of the polymer replacing them and forming water‐soluble tri‐potassium cyamelurate. Crucially, this approach enabling us to dissolve melon polymer has a potential to spectroscopically separate the contribution from species which possibly endow R‐CN_x_ with extended light‐absorption in the visible range. Notably, the dissolution of the Y‐CN_x_ and R‐CN_x_ samples resulted in colourless and yellow‐green coloured solutions, respectively (Figure 4a). The evaporation and slow cooling of these solutions lead in both cases to the formation of needle‐shaped single‐crystals of different colour (Figure4a). In spite of that, the ^13^C NMR analysis of the crystals dissolved in D_2_O shows identical signals at 158.2 and 168.7 ppm for both samples with the integrated areas ratio of ca. 1 : 1 and assigned to CN_3_ of the heptazine and N−C−O^−^ species, respectively, which is typical for potassium salts of cyameluric acid (Figures 4b and c).^[20]^ In order to evaluate the presence of minor components in the solutions, which might be responsible for the R‐CN_x_ coloration, the obtained solutes of the PCN samples in KOH were concentrated by evaporation, while the formed tri‐potassium cyamelurate crystals were removed from the system. The solution of Y‐CN_x_ sample still showed the presence of a nearly pure potassium salt of cyameluric acid (Figure 4d), having only one additional peak at 171.6 ppm, which we attribute to the C of potassium carbonate. In the ^13^C NMR spectrum of the solution of R‐CN_x_ in KOH, on the other hand, the signal ascribed to carbon atoms in CN_3_ environment of the heptazine is absent, instead the intense peaks at the chemical shifts of 168.6‐168.4 ppm suggest the presence of triazine species bearing fully deprotonated N−C−O^−^ groups^[42]^ (Figure 4e). Additionally, a peak at 173.5 ppm is observed, which can be ascribed to the presence of C−N=N−C inter‐triazine linkages (Figure 4e).^[43]^

**Figure 5:**
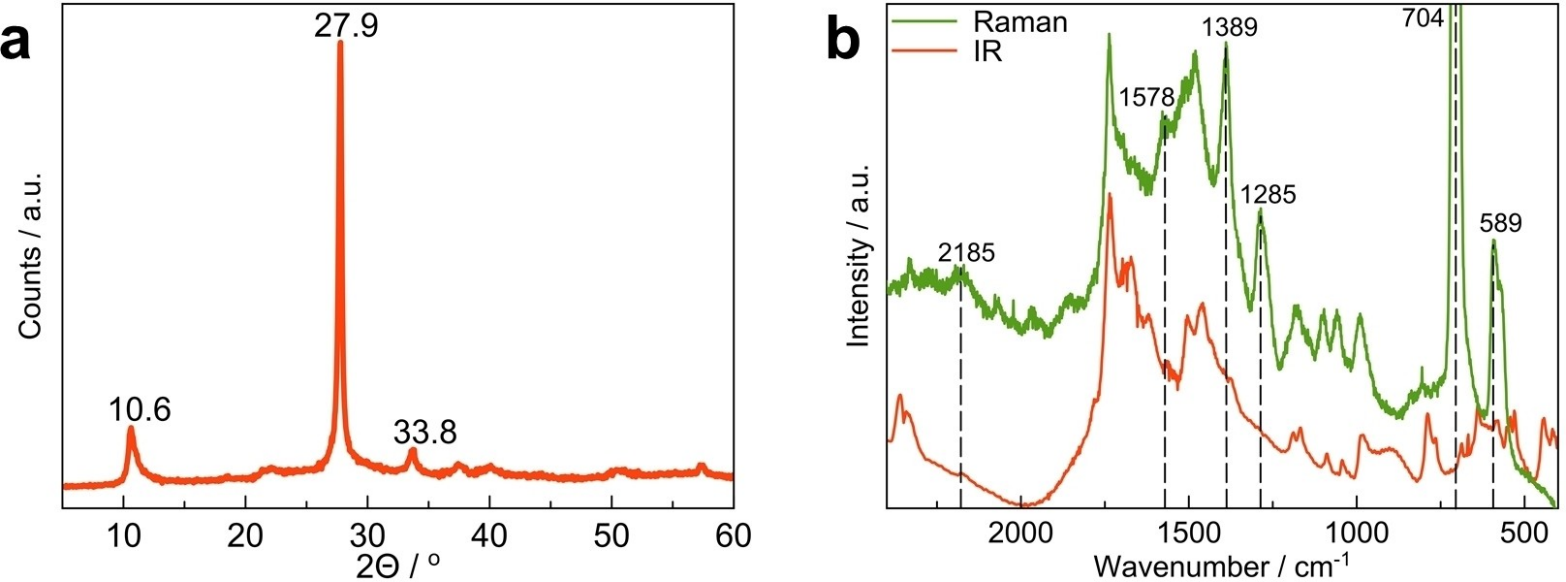
(a) XRD pattern and (b) comparative analysis of FTIR and Raman spectra of the insoluble R‐CN_x_‐KS powder obtained by slow evaporation of R‐CN_x_‐K solution.

After tri‐potassium cyamelurate crystals were extracted from the concentrated product of the R‐CN_x_ dissolution in KOH, the slow evaporation of the solution yielded a yellow powder (R‐CN_x_‐KS) with the composition C_1.00_N_1.90_O_0.90_K_0.01_, according to EDX elemental analysis, which is insoluble in water and DMSO. According to the XRD analysis (Figure 5a) and considering the presence of only triazine species in the initial solution, the crystalline phase of the separated compound might be ascribed to the poly(triazine imide) phase.^[44]^ FTIR and Raman spectra reveal a wide absorption region in 1400–1660 cm^−1^ ascribed to C=N stretching of the heterocycle and a very intense Raman band at 704 cm^−1^ accompanied by a weaker band at 989 cm^−1^ (Figure S8a,b) attributed to triazine breathing modes,^[45]^ thus corroborating the polytriazine nature of the material. The intense absorption peak at 1736 cm^−1^ coincides with the C=O bond stretching vibrations in alkali isocyanurates (Figure S8a,b), while the absorption in the 3145–3340 cm^−1^ range and at ca. 2850 cm^−1^ is attributed to free and H‐bonded NH_x_‐moieties, respectively.^[46]^ This suggests that the fully deprotonated cyanurate mono or oligomeric species in the solution undergo tautomeric transformation producing insoluble polymerized isocyanurate fragments. A comparative analysis of UV Raman and FTIR spectra shows multiple strongly Raman active modes that are not visible in the IR spectrum 1578, 1389, 1285 and 589 cm^−1^. These can be attributed to N=N stretching,^[47]^ N=N in trans‐aromatic azo compounds, azoxy groups^[34a]^ and C−N=N stretching combined with a C−N deformation vibrations,^[48]^ respectively. The observed bands at 1389 and 589 cm^−1^ are especially indicative of a *trans*‐azo moiety inside the R‐CN_x_‐KS (i. e., in the solid obtained by slow evaporation of the R‐CN_x_‐K solution that was obtained after filtering off the tri‐potassium cyamelurate crystals from the concentrated product solution of the R‐CN_x_ dissolution in KOH). Considering that it is very unlikely that azo groups are formed during KOH treatment of R‐CN_x_, we conclude that they were a part of its structural units in the solid‐state, linking the heptazine or triazine moieties and being responsible for the visible‐light absorption. Indeed, the type of the linkage between the structural units of polymeric carbon nitride determines the materials optical properties to a greater extent than the substitutional doping of the heptazine itself, as it defines the symmetry, planarity or corrugation of the polymeric system.^[49]^ Notably, a band in the visible‐light absorption region is still present in the UV‐vis spectrum of the R‐CN_x_‐KS, though blue‐shifted with respect to that observed for R‐CN_x_ (Figure S9).

To summarize, we suggest that the high‐temperature treatment of Y‐CN_x_ induces partial heptazine fragmentation accompanied by NH_3_, CNH_2_ and C_2_N_2_ fragments evolution, as previously confirmed by mass‐spectroscopic analysis.^[50]^ The produced C_2_N_2_ species can exist in several isomeric forms, such as cyanogen (N≡C−C≡N), the most thermodynamically stable one, isocyanogen (N≡C−N=C) or diisocyanogen (C=N−N=C). Diisocyanogen was detected in the products of norbornadienonazine thermolysis at temperatures exceeding 500 °C.^[51]^ This isomer should have a higher reactivity than the rest of cyanogen isomeric forms making its isolation and investigation difficult.^[52]^ Therefore, we speculate that the thermal decomposition of melon at high temperatures could yield diisocyanogen species that immediately react with the polymer backbone producing C=N−N=C linkages between the separate heptazine or triazine units, which subsequently isomerize to C−N=N−C forming the azo‐link. In other words, a PCN material with strongly extended visible light absorption is obtained that contains, apart from typical PCN structural features, also poly(heptazine/triazine) azo‐linked fragments that are chiefly responsible for the red shift of the optical absorption edge.

**Figure 6:**
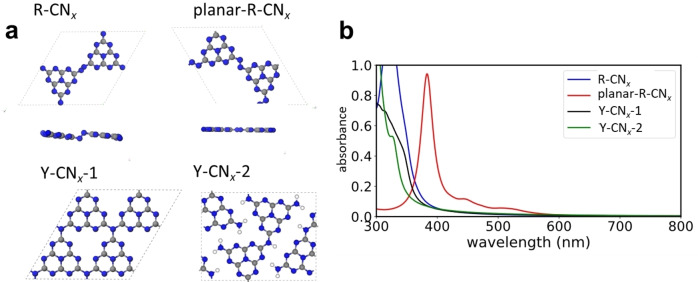
(a) Most relevant systems used for the in‐depth analysis. For the R‐CN_*x*_ systems both the top and side views are shown, while for the Y‐CN_*x*_ systems only top views are shown. (b) Calculated absorption spectra for the four different systems.

In order to better understand the electronic structures and optical properties of the different systems, periodic density functional theory calculations have been performed on various systems. Figure 6a shows the most relevant systems that were obtained after an extensive screening for possible structures of R‐CN_*x*_ and Y‐CN_*x*_. For the red carbon nitride system two different configurations were found (see Figure 6), one in a planar configuration (planar‐R‐CN_x_), and a second one with an azo‐linkage at the N=N group that leads to a step‐like structure of the layer. Interestingly, the planar configuration is 0.42 eV less stable than the R‐CN_*x*_ system (Figure S10). However, while the latter one has a bandgap of 3.5 eV, which is in the expected range of carbon nitrides, the planar‐R‐CN_x_ system has a direct bandgap of only 2.2 eV. Regarding the Y‐CN_x_, an idealized fully interconnected layer of g‐C_3_N_4_ ^[1c]^ (*E*_gap_=2.8 eV) as well as a melon‐type polymer with hydrogenated amino end groups (*E*_gap_=3.6 eV) that leads to strand‐like structures were considered. All band structures and projected density of states of the different systems can be found in the Supporting Information (Figure S10, S11), together with the charge density distributions at the corresponding valence band maxima (VBM) and conduction band minima (CBM) (Figure S12). The Figure 6b shows the calculated absorption spectra, which reveal the expected UV‐absorbance for all CN_*x*_ structures, including the hypothetical fully condensed g‐C_3_N_4_, except for planar‐R‐CN_*x*_ that shows absorption at longer wavelengths close to 400 nm.

**Figure 7:**
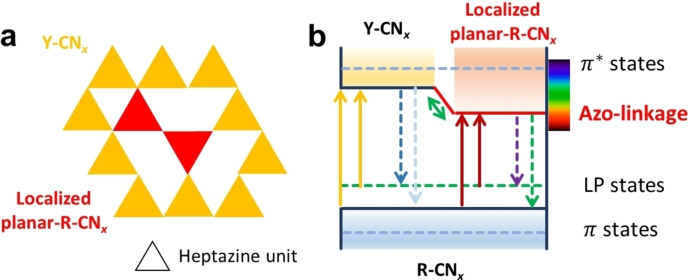
Proposed diagram of possible transitions for R‐CN_*x*_. The model (a) shows the confined localized planar‐R‐CN_*x*_ unit in the Y‐CN_*x*_ framework, while the energy diagram (b) shows the corresponding energy states. Here the azo‐linkage lowers the bandgap and leads to light absorption at wavelengths in the visible spectral range.

The question remains why the experimentally observed optical properties of red carbon nitride conforms with the calculated behaviour of planar‐R‐CN_*x*_, though this structure is less stable than the step‐like R‐CN_x_ configuration (see Figure 6). Our present hypothesis is that red carbon nitride is confined in a Y‐CN_x_ framework (as shown in Figure 7a), which stabilizes the planar configuration, leading to localized planar‐R‐CN_*x*_ units. The azo‐linkages in the planar‐R‐CN_*x*_ units then lead to reduction of the overall bandgap as depicted in the energy scheme shown in Figure 7b. Such confinement effects have also been observed for other two‐dimensional systems (e. g., TaSe_2_), where the embedding in a larger framework leads to a stabilization of the usually less preferred configuration.^[53]^

### Photoluminescence spectroscopy

**Figure 8:**
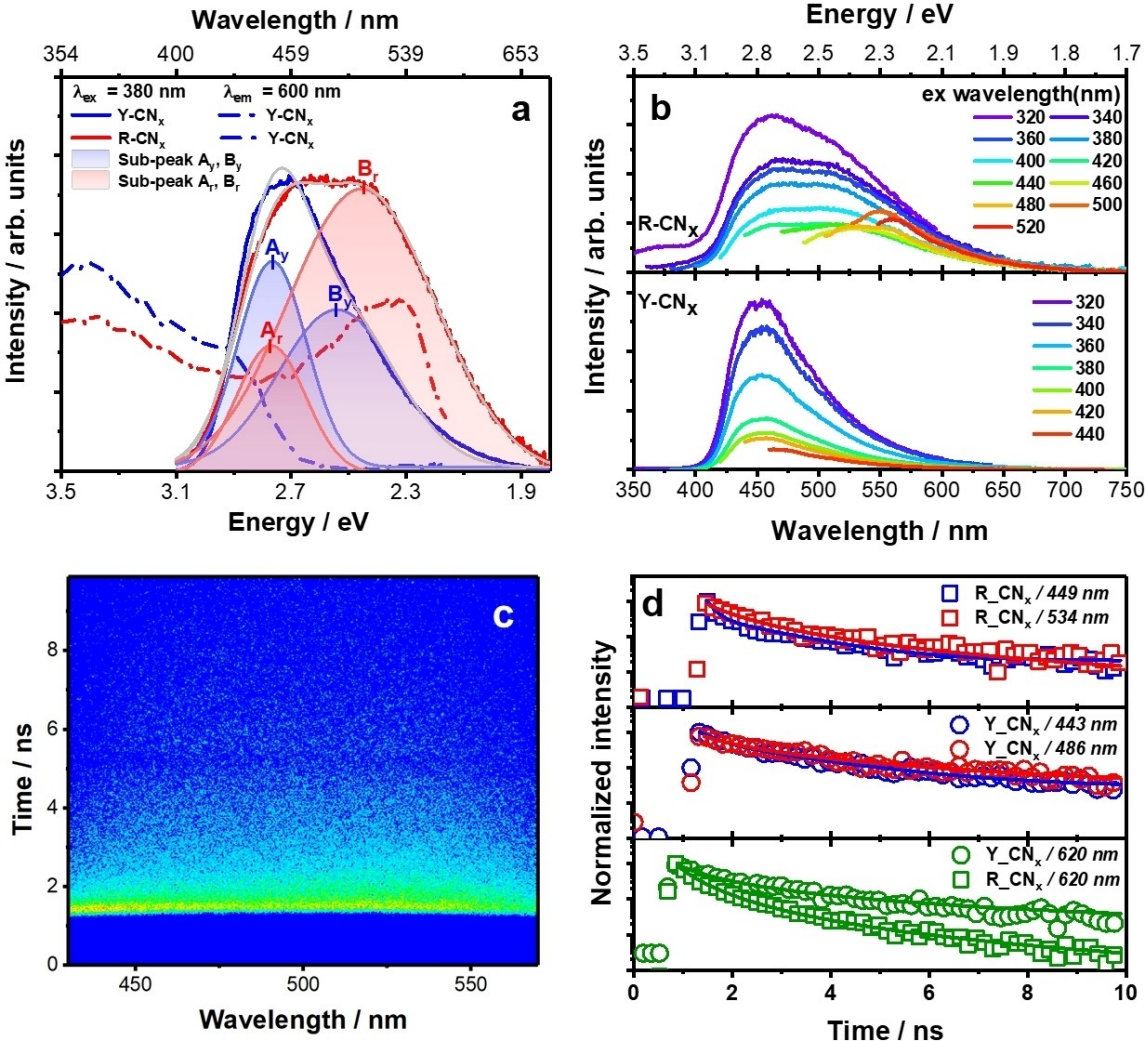
(a) PL and PL excitation spectra of drop casted Y‐CN_x_ and R‐CN_x_. Emission upon excitation at 380 (solid lines) and PL excitation spectra recorded at 600 nm (dashed lines) spectra were taken at room temperature. Multi‐peaks of the PL spectra for Y‐CN_x_ (A_y_, B_y_) and R‐CN_x_ (A_r_, B_r_) were fitted with a Gaussian function. (b) PL spectra of Y‐CN_x_ and R‐CN_x_ acquired at different excitation wavelengths. (c) Streak camera image showing the PL as a function of emission wavelength and time recorded for drop casted R‐CN_x_ upon 385 nm excitation. The experimental time window was chosen to be 10 ns. (d) PL decay kinetics of R‐CN_x_ after excited at 385 nm (upper panel) and 520 nm (lower panel). To derive the kinetics, the streak camera data has been integrated in a spectral range of ±5 nm with respect to the probe wavelengths indicated in panel (d). For comparison the respective PL decay kinetics of Y‐CN_x_ are shown.

Photoluminescence (PL) spectra of drop‐casted CN_x_ films are shown in Figure 8a. The PL of R‐CN_x_ is spectrally broader and appears red‐shifted compared to the benchmark material Y‐CN_x_. Fitting the steady‐state PL spectra of both materials to a sum of two Gaussian peaks reveals that the apparent spectral broadening of the R‐CN_x_ spectrum is due to the increase of a low‐energy emission component (sub‐peak B) below 2.5 eV. When monitoring the low‐energy emission of R‐CN_x_, a distinct new peak (centered at 540 nm) becomes apparent in the PL excitation spectrum. This suggests the presence of distinct emissive states below the band edge of R‐CN_x_,^[18]^ which are clearly visible also in the PL spectra recorded for different excitation wavelengths (Figure 8b). The sub‐bandgap emission observed in R‐CN_x_ is likely associated with an increased number of tail states due to the higher density of localized planar‐R‐CN_*x*_ units in the Y‐CN_*x*_ framework (Figure 7) produced by heptazine fragmentation and azo‐linkages formation after elevated temperature processing.^[54]^

To obtain more insight into the distinct PL properties of R‐CN_x_, time‐resolved PL data were collected upon excitation at 380 and 520 nm. The choice of excitation wavelengths enables a comparison between bandgap excitation and direct excitation of the sub‐band emission. Figure 8c exemplarily shows the streak camera data for R‐CN_x_ upon excitation at 385 nm. The transient PL spectra can be fitted to a sum of two Gaussian peaks centered at 2.32 and 2.76 eV (Figure S13a). The corresponding time‐resolved PL spectra for Y‐CN_x_ yield emission contributions at 2.55 and 2.80 eV (see Figure S13a). Upon excitation at 520 nm a long‐wave PL emission centered at 2.20 eV is visible (Figure S13b). To derive PL kinetics reflecting the lifetime of the emission upon excitation at 385 nm, the transient PL spectra are integrated in over 10 nm around the peak wavelength of the individual emission contributions (Figure 8d). The transient PL kinetics of R‐CN_x_ (recorded at about 2.8 eV) are fitted by a bi‐exponential decay yielding the characteristic time constants τ_1_=0.17 ns and τ_2_=1.34 ns. For Y‐CN_x_ the equivalent data decay with slightly longer time constants, i. e. τ_1_=0.39 ns and τ_2_ =1.91 ns. This comparison indicates somewhat faster exciton dissociation in R‐CN_x_, likely associated with higher defect density and the more localized excited state in the planar R‐CN_x_ units (see Figure 7). The bi‐exponential decay of the excitonic emission is related to different recombination centres in the material. If excitons are generated in proximity to such a recombination centre, they decay more rapidly on a sub‐ns timescale, while excitons generated distant from such recombination centres decay on a longer timescale. The decay of the sub‐bandgap emission (recorded upon excitation at 520 nm and derived by integration over the entire PL band) is non‐exponential decay, i. e. it follows a power law kinetic $I_{PL}\left(t\right)∼t^{\;-\alpha }$ commonly used to account for reaction kinetics in diffusive systems.[^55^, ^56^] Comparing R‐CN_x_ to Y‐CN_x_, the value of the characteristic exponent decreases from $\alpha_{rCNx}\approx 2.2$ to $\alpha_{yCNx}\approx 1.3$ . The more rapid decay in R‐CN_x_ is attributed to an increased number of defect states serving as recombination centres.

### Photocatalytic hydrogen evolution

**Figure 9:**
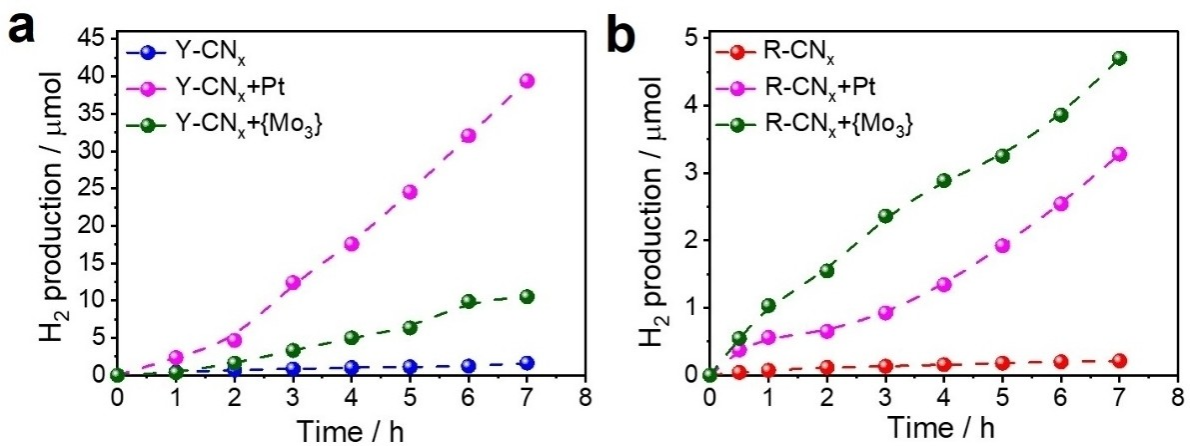
Photocatalytic HER performed using (a) Y‐CN_x_ and (b) R‐CN_x_ based materials under monochromatic 420 nm LED irradiation. Conditions: [catalyst]: 10 mg, Solvent: H_2_O:MeOH (9 : 1, v : v), in absence of any additional electron donor.

Conventional Y‐CN_x_ modified with appropriate co‐catalysts is known for its ability to produce H_2_ in presence of a sacrificial reducing agent under visible light irradiation.^[1a]^ Pristine Y‐CN_x_ is inactive for photocatalytic HER under monochromatic LED light irradiation at 420 nm (Figure 9a). However, after deposition of the {Mo_3_} clusters and especially Pt nanoparticles, it demonstrates a very good performance reaching a H_2_ production of around 0.04 mmol in 7 h (Figure 9a). Although the experimental data exposed and discussed above indicate that the basic structure of conventional yellow PCN is preserved also in the R‐CN_x_ material, its photocatalytic behaviour is very different. The activity of the red R‐CN_x_ modified with {Mo_3_} clusters or Pt nanoparticles is lower by an order of magnitude compared to Y‐CN_x_, reaching only 0.005 mmol H_2_ after 7 h of irradiation (Figure 9b). Similar to the conventional PCN counterpart, bare R‐CN_x_ without a co‐catalyst does not photocatalysed HER under 420 nm light irradiation (Figure 9b). Contrary to the case of Y‐CN_x_, R‐CN_x_ shows higher photocatalytic activity if modified with the {Mo_3_} clusters than with Pt nanoparticles (Figure 9b). A similar observation was reported by Wang et al. for protonated mesoporous carbon nitride materials.^[57]^ These results suggest that the surface properties of the R‐CN_x_ sample are different to a significant extent from those of Y‐CN_x_, affecting the interaction of the former with co‐catalysts.

**Figure 10:**
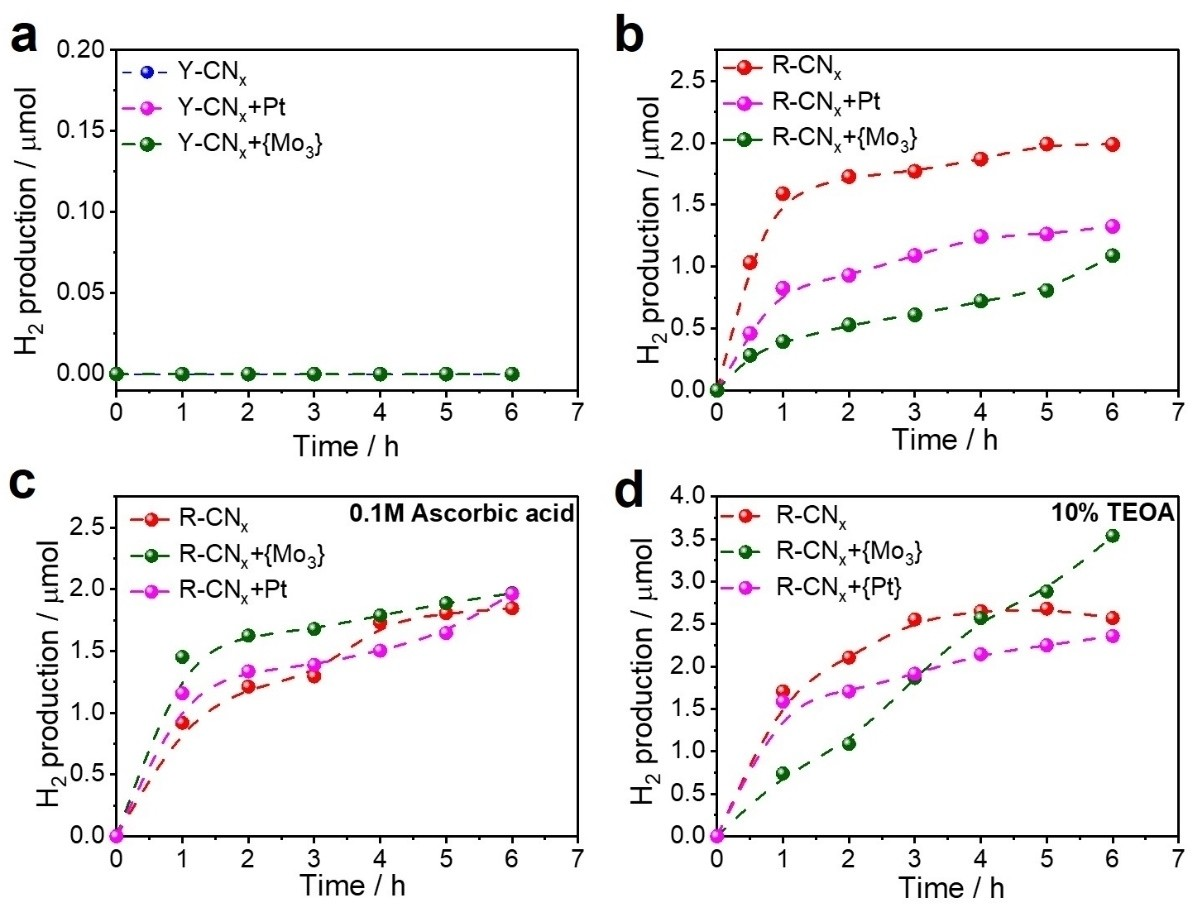
Photocatalytic HER performed using (a) Y‐CN_x_ and R‐CN_x_ based materials under 470 nm irradiation (b) in absence of any electron donor, (c) in presence of ascorbic acid (0.1 M; pH 2.4) and (d) 10 % of TEOA (pH 10.5). Conditions: [catalyst] : 10 mg, Solvent: H_2_O:MeOH (9 : 1, v : v).

The photocatalytic activity of R‐CN_x_ under 420 nm irradiation is inferior to that of its yellow counterpart, nonetheless the appearance of an additional absorption band in the visible‐light range in the UV‐vis spectrum of R‐CN_x_ (Figure 1b and d) prompted us to study its performance also under excitation with lower energy light at the wavelength of 470 nm. Under these conditions, the Y‐CN_x_ sample is, as expected, completely inactive in photocatalytic HER both with and without co‐catalysts (Figure 10a), since it does not absorb in this spectral region (Figures 1b and c). Intriguingly, the red R‐CN_x_ is not only capable of sacrificial H_2_ evolution under irradiation at 470 nm when modified with {Mo_3_} or Pt nanoparticles, but the pristine R‐CN_x_ without any co‐catalysts even shows a superior photocatalytic activity (Figure 10b). Therefore, the new functional groups in the red carbon nitride, the azo‐linked poly(heptazine/triazines) formed as the result of the high‐temperature treatment, are not only responsible for the strong red shift of the optical absorption edge of the material, but they also might possibly play an additional role of an active site for H_2_ evolution. Despite the rather moderate activity of this material, its ability to absorb a significant portion of the visible light spectrum and the metal‐free nature of its active site for photocatalytic HER represent intriguing aspects of R‐CN_x_ with respect to solar light‐driven photocatalysis. From the R‐CN_x_ catalysed H_2_ evolution plots one can observe an apparent deactivation trend (Figure 10b).

We hypothesized that this was due to the weak reductive power of methanol as a sacrificial reducing agent, hence some stronger reductants were added to the R‐CN_x_ suspensions. The addition of ascorbic acid improved the photocatalytic performance of R‐CN_x_ modified with {Mo_3_} and Pt nanoparticles up to the level of the pristine catalyst, while leaving the activity of the co‐catalyst modified samples unchanged (Figure 10c). The introduction of another strong reductant, triethanolamine (TEOA), affected the activity of bare R‐CN_x_ only slightly, but had a strong impact on the activity of co‐catalyst‐modified R‐CN_x_ (Figure 10d). Most notably, the strong deactivation trend could be reversed for the {Mo_3_}‐modified R‐CN_x_ photocatalyst in the presence of TEOA, leading to a stable rate of H_2_ evolution (green trace in Figure 10d). This might be attributed to a more effective oxidation of TEOA compared to ascorbic acid since positively charged TEOA at pH 10.5 (pK_a_=7.9) is expected to adsorb more effectively at the negatively charged R‐CN_x_ than non‐dissociated ascorbic acid (pK_a_=4.3) at pH 2.4.

## Conclusions

We report a simple post‐synthetic approach involving a brief high‐temperature treatment under vacuum that effectively turns the typically yellow PCN into a red PCN by extending significantly its visible light absorption to >600 nm. Most importantly, we provide evidence that the extended optical absorption edge of PCNs subjected to high‐temperature treatment is due to the formation of azo‐moieties that act as linkages between the heptazine units inherent to PCN structure and/or between heptazine and triazine units formed upon partial fragmentation of PCN. Our present understanding is that the formation of planar‐R‐CN_*x*_ units comprising the azo‐linkages and embedded in the yellow carbon nitride framework is responsible for the reduction of the overall bandgap and the red colour of the material. Notably, the azo moieties effectively sensitize the red PCN for photocatalytic H_2_ evolution even under low energy (470 nm) light where conventional yellow PCN is inactive. Moreover, the red carbon nitride clearly contains moieties that can act as metal‐free catalytic sites for H_2_ as evidenced by effective operation even without any additional co‐catalyst. The downside of the high‐temperature treatment of PCN is the partial fragmentation of the heptazine network creating large number of recombination centres and decreasing the overall photocatalytic activity of the resulting photocatalyst. We assume that our findings on the so far overlooked role of azo moieties in PCNs with strongly extended absorption edge might not only be valid for this particular case, but also for other cases in which a strong shift of the PCN absorption edge has been observed upon thermal treatment procedures at temperatures leading to the heptazine fragmentation and formation of cyanogen species. The present work thus highlights the importance of a careful determination of structural features governing the complex interplay between the light absorption, charge separation and catalytic turnover in PCN‐based polymers tailored for visible light‐driven photocatalysis.

## Conflict of interest

The authors declare no conflict of interest.

## Supporting information

As a service to our authors and readers, this journal provides supporting information supplied by the authors. Such materials are peer reviewed and may be re‐organized for online delivery, but are not copy‐edited or typeset. Technical support issues arising from supporting information (other than missing files) should be addressed to the authors.

Supporting Information
