# Supplementary material for: A Study in Red: The Overlooked Role of Azo‐Moieties in Polymeric Carbon Nitride Photocatalysts with Strongly Extended Optical Absorption
Source: Chemistry. 2021 Oct 21;27(68):17188–202. doi: 10.1002/chem.202102945 (PMC9298046; doi:10.1002/chem.202102945)
Supplement: Supplementary file 1 — Supporting Information [file CHEM-27-17188-s001.pdf]

Chemistry–A European Journal

Supporting Information

A Study in Red: The Overlooked Role of Azo-Moieties in Polymeric Carbon Nitride Photocatalysts with Strongly Extended Optical Absorption

Dariusz Mitoraj, Igor Krivtsov,* Chunyu Li, Ashwene Rajagopal, Changbin Im, Christiane Adler, Kerstin Köble, Olena Khainakova, Julian Hniopek, Christof Neumann, Andrey Turchanin, Ivan da Silva, Michael Schmitt, Robert Leiter, Tibor Lehnert, Jürgen Popp, Ute Kaiser, Timo Jacob, Carsten Streb, Benjamin Dietzek,* and Radim Beranek*

SUPPORTING INFORMATION

A Study in Red: The Overlooked Role of Azo-Moieties in Polymeric Carbon Nitride Photocatalysts with Strongly Extended Optical Absorption

Dariusz Mitoraj, Igor Krivtsov, Chunyu Li, Ashwene Rajagopal, Changbin Im, Christiane Adler, Kerstin Köble, Olena Khainakova, Julian Hniopek, Christof Neumann, Andrey Turchanin, Ivan da Silva, Michael Schmitt, Robert Leiter, Tibor Lehnert, Jürgen Popp, Ute Kaiser, Timo Jacob, Carsten Streb, Benjamin Dietzek,* Radim Beranek**

*Corresponding authors: igor.krivtsov@uni-ulm.de, benjamin.dietzek@uni-jena.de, radim.beranek@uni-ulm.de

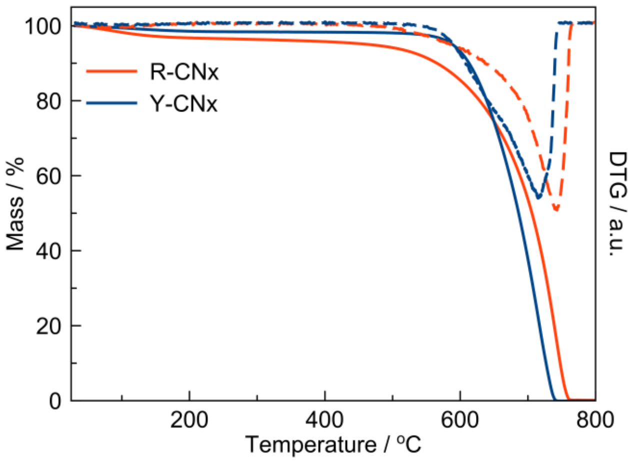

Figure S1. Thermogravimetric analysis of the Y-CN_x and R-CN_x samples

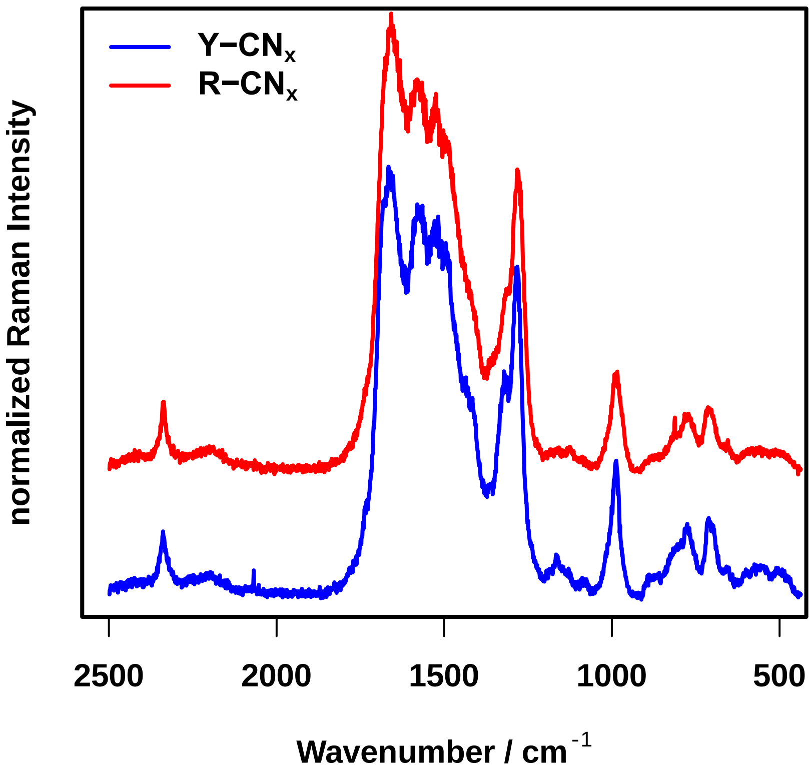

Figure S2. UV-Raman spectra of the Y-CN$_x$ and R-CN$_x$ samples

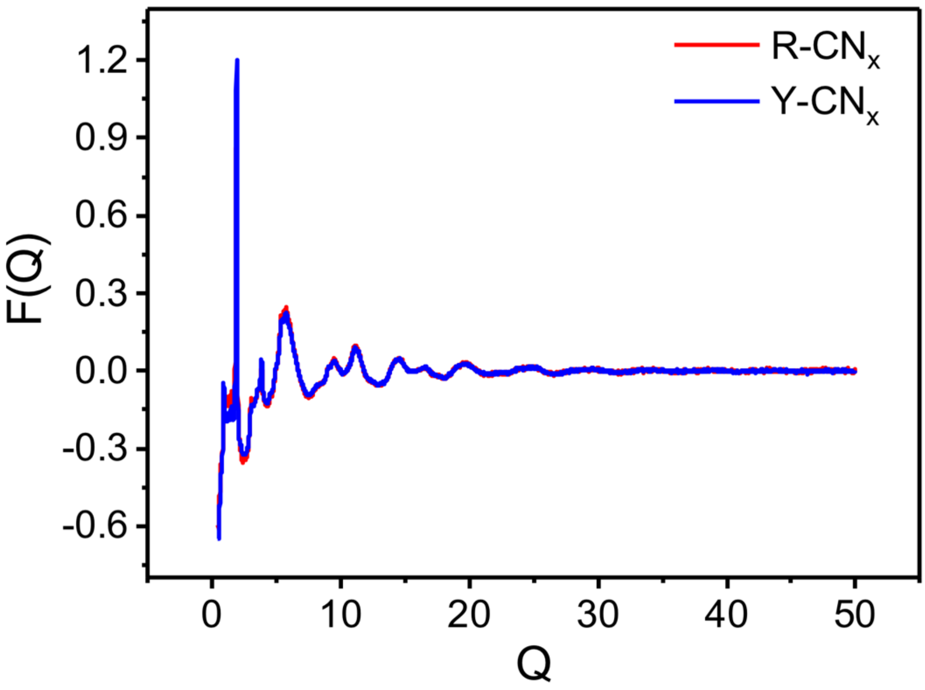

Figure S3. Neutron diffraction structure function obtained for Y-CN$_x$ and R-CN$_x$

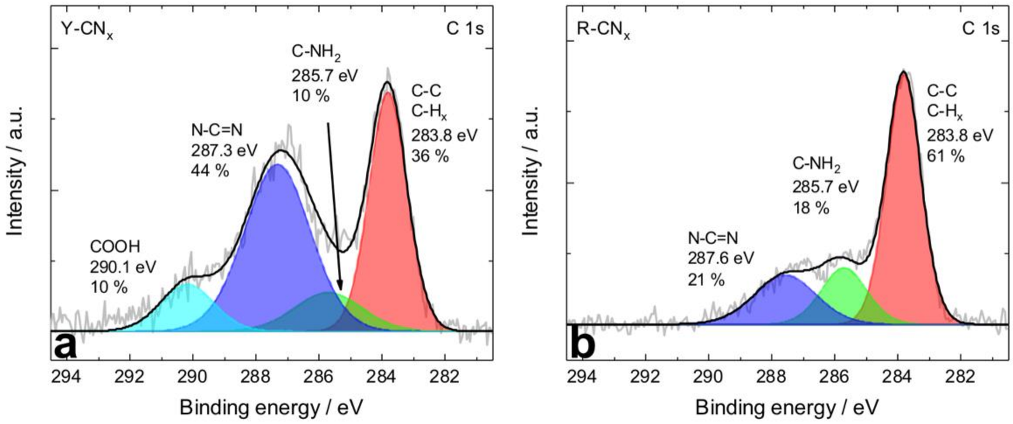

Figure S4. XP C 1s spectra of the Y-CN$_x$ (a) and R-CN$_x$ (b) samples

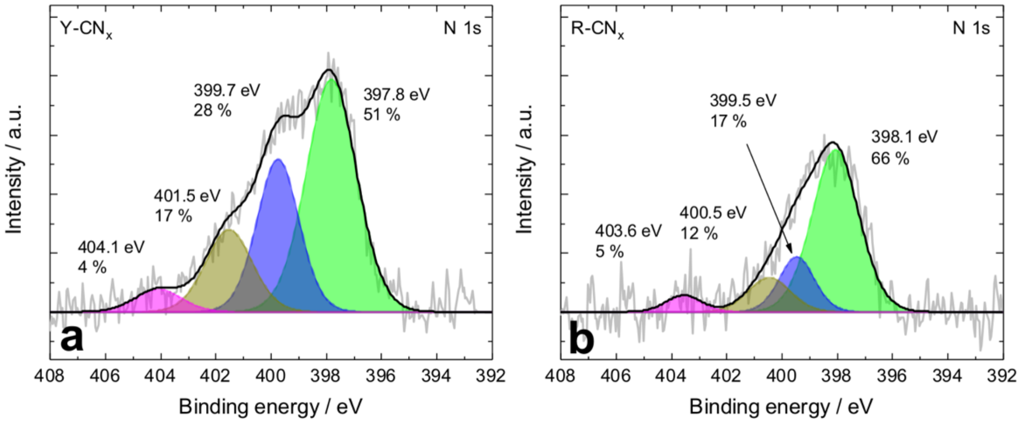

Figure S5. XP N 1s spectra of (a) Y-CN$_x$ and (b) R-CN$_x$ samples

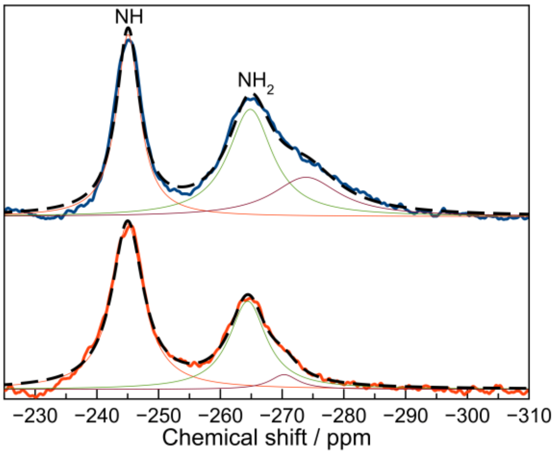

Figure S6. Deconvoluted ^{1}H-^{15}N SS NMR spectra of the Y-CN$_x$ and R-CN$_x$ samples

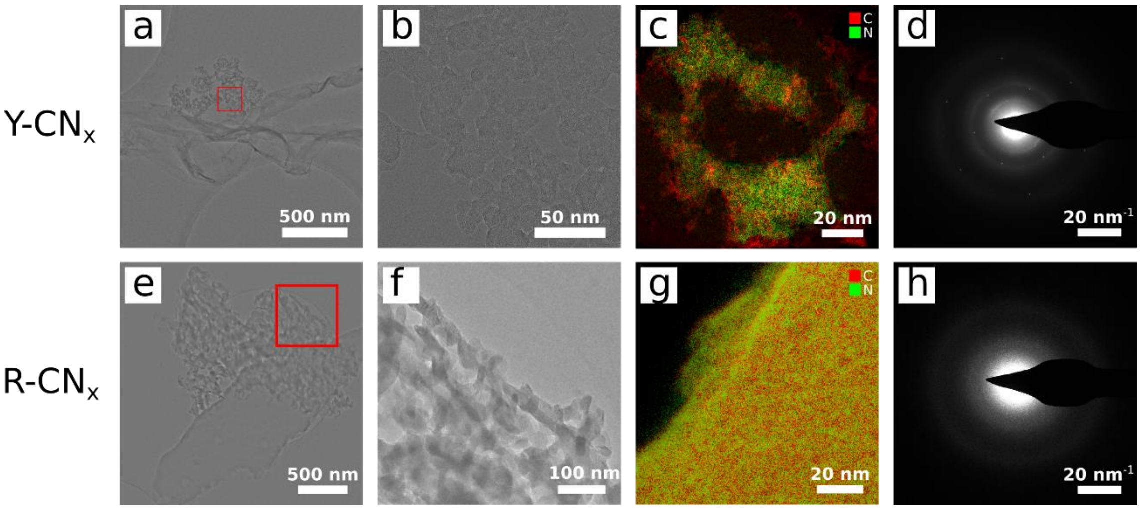

Figure S7. TEM data for Y-CN_x (a-d) and R-CN_x (e-h): Overview image (a) of the sample on amorphous carbon film with graphene as a support. The red marked area is further magnified in (b) showing agglomerated particles. Energy filtered TEM (EFTEM) mapping (c) of C- and N-K lines show homogeneous distribution of carbon and nitrogen in the particles. Electron diffraction pattern (d) shows amorphous rings in addition to the graphene spots. Overview image (e) for R-CNx shows similar agglomerations, further magnified from the red marked area in (f). EFTEM mapping (g) from the region shows homogeneous distribution of C and N. Diffraction pattern (h) shows amorphous rings.

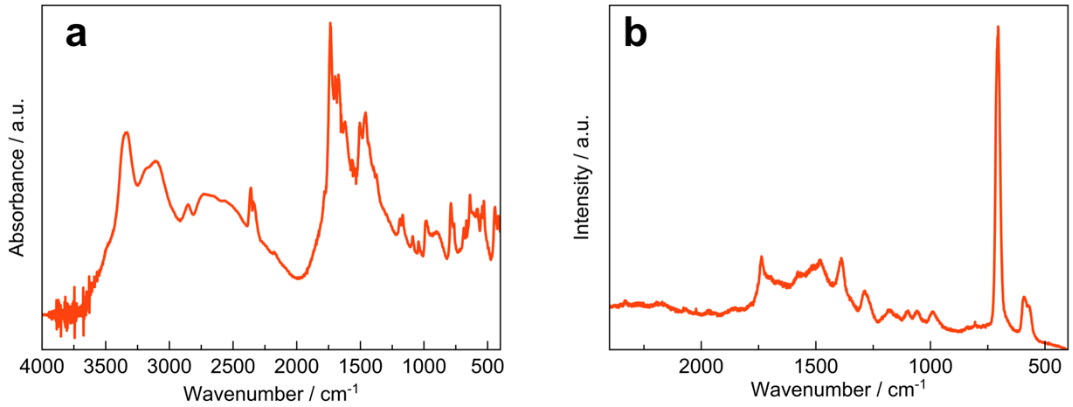

Figure S8. (a) FTIR and **(b)** Raman spectra of the R-CN_x-KS sample

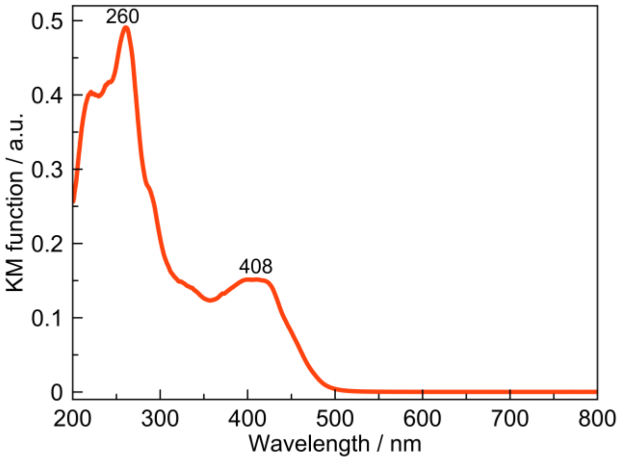

Figure S9. DRUV-vis spectrum of the R-CN_x-KS sample

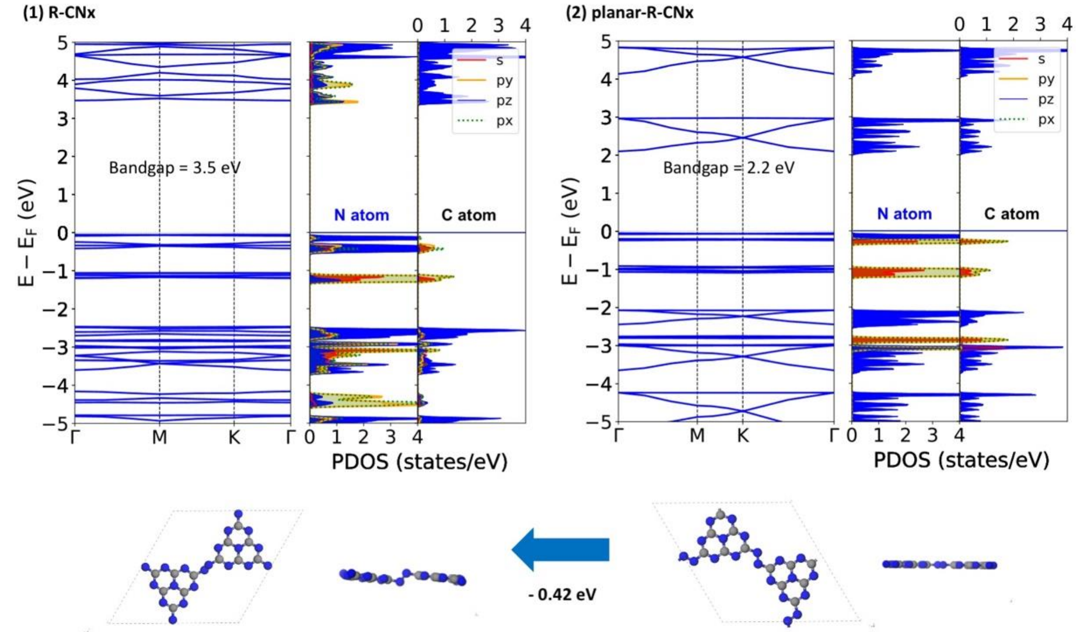

Figure S10. Electronic band structures, projected density of states and the structural images of the R-CN_x models.

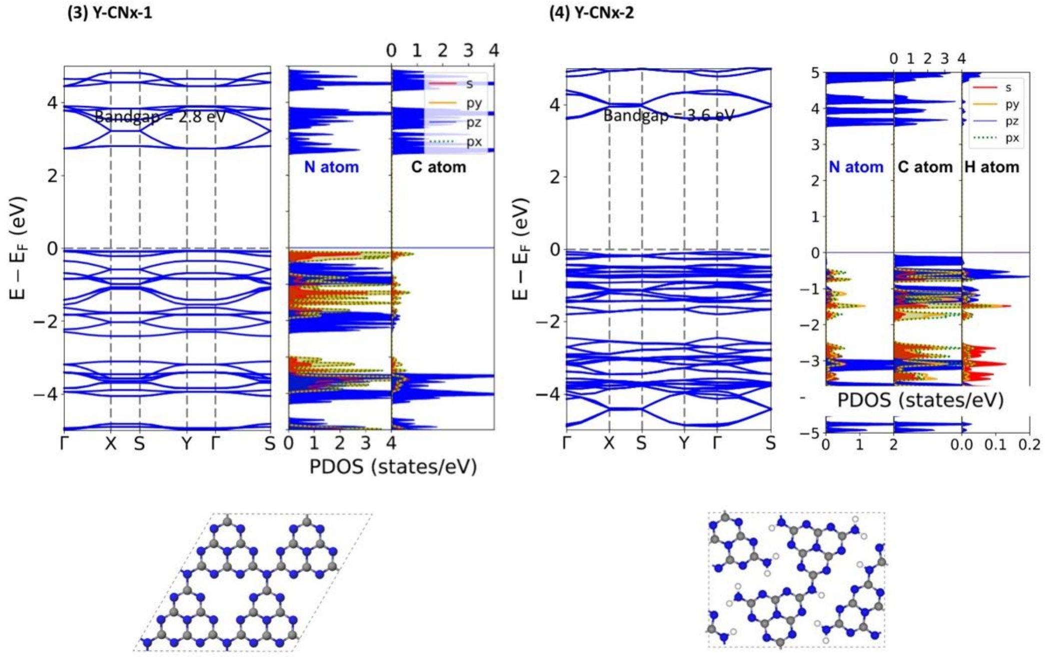

Figure S11. Electronic band structures, projected density of states and the structural images of the Y-CN_x models.

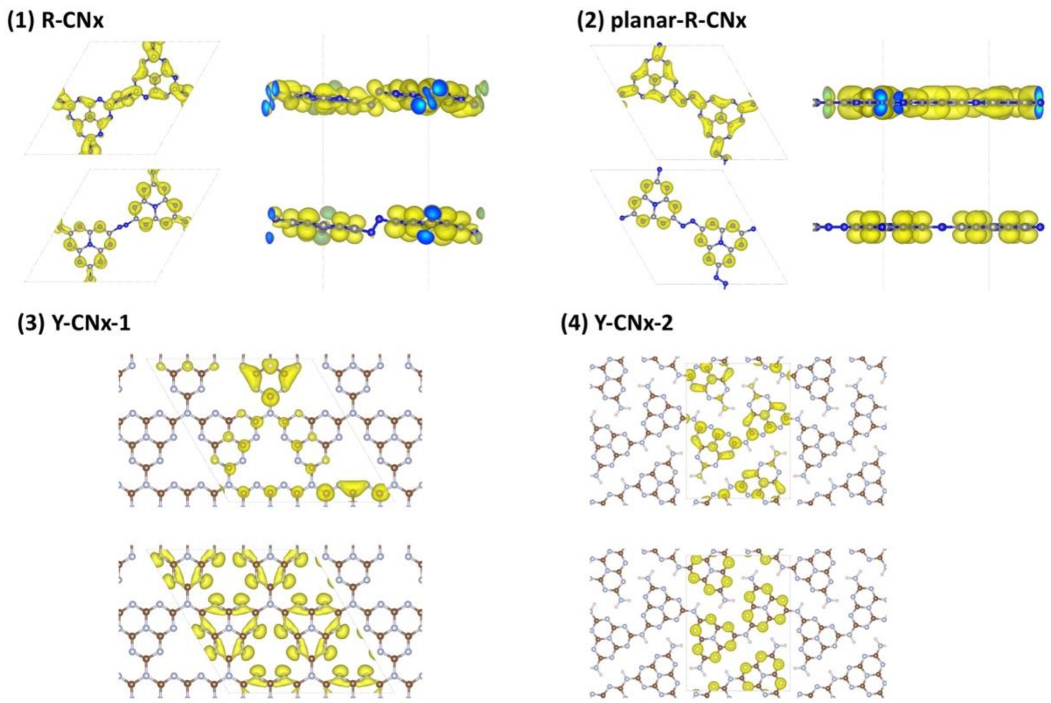

Figure S12. Charge densities at VBM (bottom) and CBM (top). The blue color is for nitrogen and the silver is for carbon.

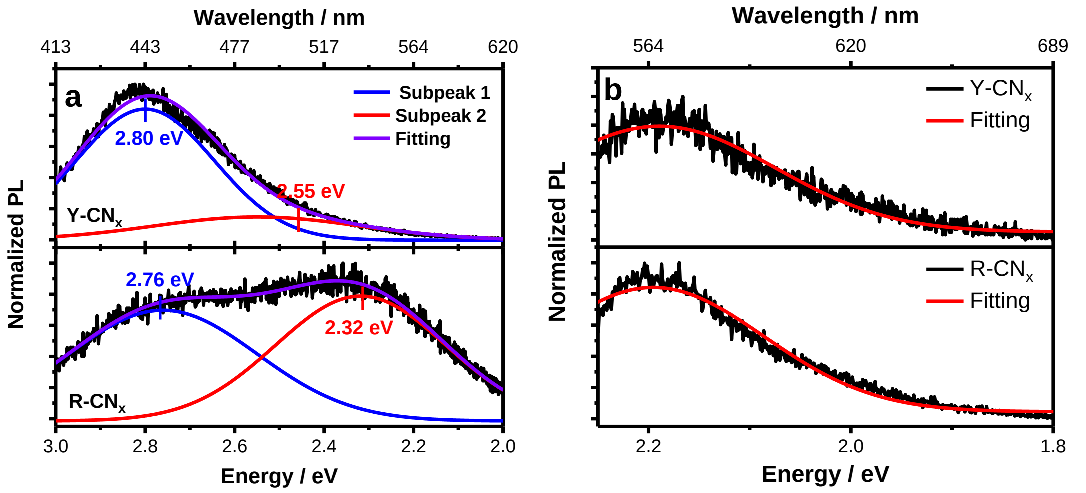

Figure S13. Gaussian fit of time-resolved emission spectra of dropcasted Y-CN_x and R-CN_x under 385 nm (a) with the high energy (blue line) and low energy (red line) PL centers and under 520 nm excitation.
